# Objectively measured physical activity and sedentary time in children with overweight, obesity and morbid obesity: a cross-sectional analysis

**DOI:** 10.1186/s12889-021-11555-5

**Published:** 2021-08-17

**Authors:** Gabrielle ten Velde, Guy Plasqui, Elke Dorenbos, Bjorn Winkens, Anita Vreugdenhil

**Affiliations:** 1 Centre for Overweight Adolescent and Children’s Healthcare (COACH), Maastricht University Medical Centre, P.O. Box 5800, 6202 AZ Maastricht, the Netherlands; 2 Department of Nutrition and Movement Scienes, Maastricht University, P.O. Box 616, 6200 MD Maastricht, The Netherlands; 3 Department of Methodology and Statistics, Care And Public Health Research Institute (CAPHRI), Maastricht University, P.O. Box 616, 6200 MD Maastricht, The Netherlands

**Keywords:** Childhood obesity, Morbid obesity, Physical activity, Accelerometer

## Abstract

**Background:**

Limited physical activity (PA) and a high level of sedentary time (ST) are associated with childhood obesity and are a target for intervention. This study aimed to assess objectively measured PA and ST in Dutch children across weight categories, age groups and sex.

**Methods:**

202 children with overweight, obesity and morbid obesity (55% girls, 12 ± 3y of age, BMI z-score + 3.15 ± 0.73), referred to the Centre for Overweight Adolescents and Children’s Healthcare (COACH, Maastricht UMC+) were included. PA (total PA, light PA and moderate to vigorous PA (MVPA)) and ST were measured with the GT3X Actigraph accelerometer. Wear time validation was set to include at least four days, 480 min/day, including one weekend day.

**Results:**

PA levels in children with morbid obesity were higher compared to children with obesity, also after correction for age and sex (corrected difference (B) 118 counts per minute (cpm), *p* = .006). ST was lower in children with morbid obesity compared to children with obesity (B − 51 min/day, *p* = .018). Girls performed significantly less MVPA than boys (B − 11 min/day, *p* < .001) and for each year increase of age, children performed less PA (B − 46 cpm, p < .001) and ST increased (B 18 min/day, p < .001).

**Conclusion:**

PA and ST is different in subgroups of children with overweight, obesity and morbid obesity, depending on sex, age and overweight severity. In particular, children with obesity perform less PA and more ST compared to children with morbid obesity. Future research could explore the preferences and needs for PA and ST in children in the different weight categories.

**Trial registration:**

The trial is registered with Clinicaltrials.govNCT02091544 at March 19, 2014.

**Supplementary Information:**

The online version contains supplementary material available at 10.1186/s12889-021-11555-5.

## Background

Over the last few decades, childhood overweight and obesity rates have increased globally. In 2020, in the Netherlands, the prevalence of overweight in primary school children (4–12 year-old) and adolescents (12–16 year-old) was 13.2 and 19.3% respectively, and of obesity 2.7 and 2.3% [1] . The prevalence of Dutch children with morbid obesity was 0.59% in boys and 0.53% in girls in 2009 [2]. Alarmingly, morbid obesity is the fastest growing subcategory of childhood obesity worldwide [2, 3]. The shift towards more severe forms of childhood obesity is associated with an increased health risk for developing life-threatening chronic diseases, psychological disorders and premature death [2, 4, 5]. Overweight in childhood often tracks into adulthood, further increasing the risk of comorbidities [6–8].

Low levels of physical activity (PA) and a high amount of sedentary time (ST) play an important role in the development of childhood overweight and progression to more severe obesity [9]. Guidelines have been developed to promote PA and reduce ST to foster health benefits [10]. The World Health Organization, as well as the Dutch government, recommends that children and adolescents spend a minimum of 60 min of moderate to vigorous physical activity (MVPA) each day, preferably including more vigorous intensity activities and ST should be minimized [10–12]. Subjectively measured data indicates that 45% of Dutch children (4–11 year-old) and 69% of adolescents (aged 12–18 years) do not meet these public health guidelines [1]. Even though these guidelines have been developed internationally, the evaluation of PA levels and ST specifically among children with overweight and (morbid) obesity is lacking. Therefore, also insight in the potential for PA improvement and ST reduction as a target for intervention in these subgroups is missing.

The study of Salawi et al. (2014) included children (6–19 years) with morbid obesity and showed that these children self-reported to perform on average 18 min less MVPA compared to children with overweight or obesity (51 vs 69 min/day respectively) [13]. Unfortunately, ST was not evaluated in that study. However, subjective methods, such as the questionnaires used in the study of Salawi et al. (2014), tend to overestimate levels of PA. Objective measurements, such as accelerometry, are more accurate to determine the amount of PA and ST [14]. A review of Elmesmari et al. (2018) included studies that used accelerometry to measure PA and ST and reported that these studies in general showed that children and adolescents with obesity are significantly less physically active and more sedentary compared to children without obesity [15]. Though, in all of these studies PA and ST were not compared between children with different degrees of overweight and obesity. Furthermore, influencing factors such as age and sex should be considered when investigating PA and ST between weight categories, since it has been shown that in the general population boys are more physically active than girls and PA declines with aging [16, 17]. Only a few studies that used accelerometry included children with overweight and obesity and also investigated the effect of age and sex (17–19). For instance, the study of Jago et al. (2019) showed that in the age range 6–11 years PA decreases and ST increases with increasing age, in children with overweight and obesity (17). However, no differentiation was made for weight categories and therefore no conclusions on PA of children with morbid obesity compared to other weight categories can be made based on this study.

In summary, although it is generally accepted that the development of overweight and (morbid) obesity is multifactorial, PA and ST play an important role herein. To date, no studies have evaluated the differences between objectively measured PA and ST in children with different overweight categories. Insight in PA levels and ST in different subgroups might help to develop or improve tailored childhood obesity interventions. Therefore, the current study aimed to assess objectively measured PA (total PA, light PA and MVPA) and ST across different weight categories, age groups, and sex in Dutch children with overweight, obesity and morbid obesity. We hypothesized that PA would decrease and ST would increase with increasing overweight severity, with a sex difference in favor for boys and a negative effect of an older age in all groups.

## Methods

### Setting and participants

**Fig. 1:**
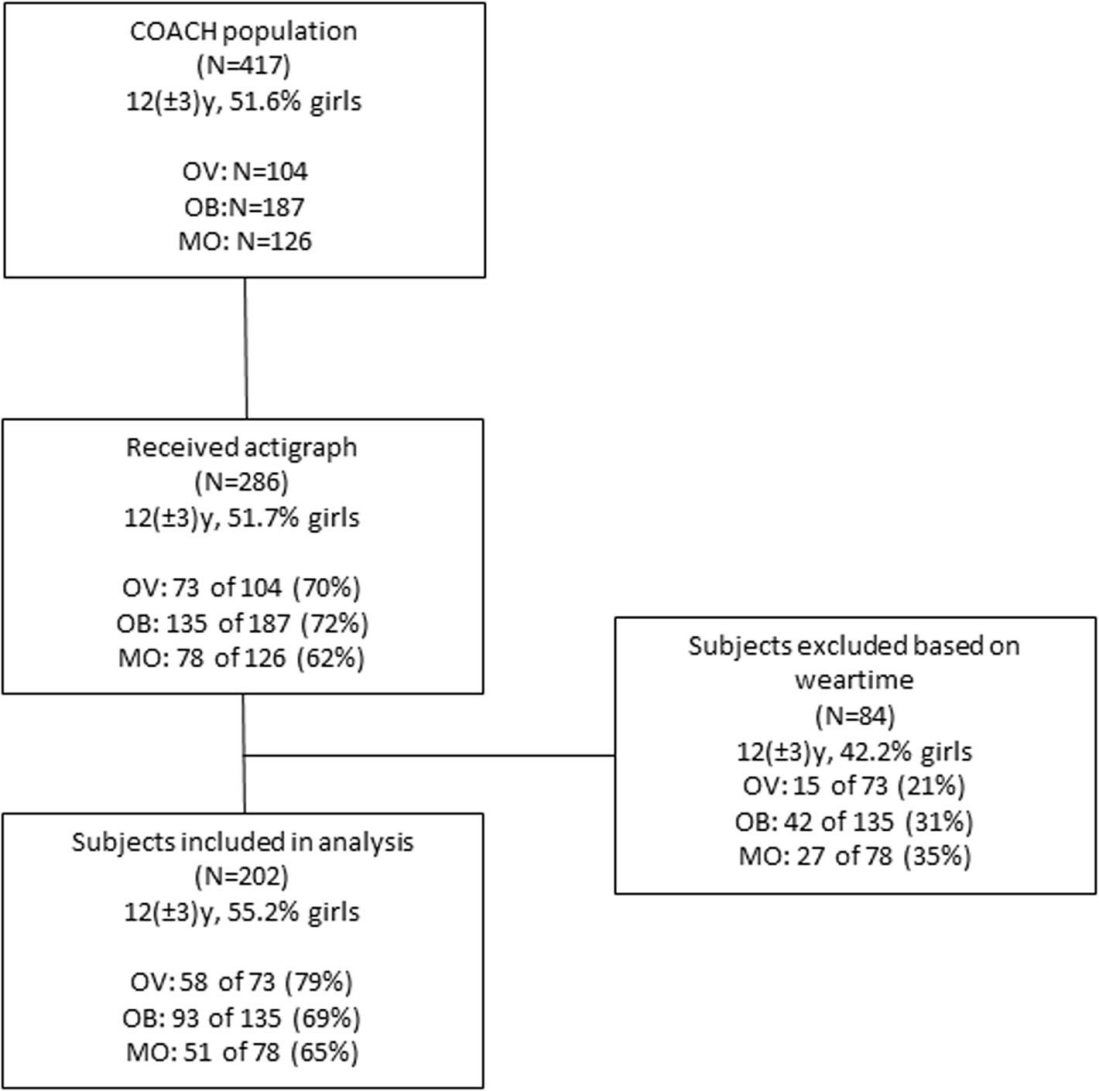
flowchart of study enrolment. Abbreviations: OV overweight; OB obese; MO morbid obese

This study was designed and conducted within the setting of the Centre for Overweight Adolescent and Children’s Health Care (COACH) at the Maastricht University Medical Centre (Maastricht, the Netherlands). Children were referred to COACH by the youth healthcare division and general practitioners. Children and their families are referred to COACH for evaluation of their physical condition and lifestyle and for individual guidance with focus on lifestyle changes as published previously [18]. There was a continuous inflow of children to the COACH program. The waiting time for the intake after referral was a maximum of 4 weeks. The present study involves a cross-sectional analysis of PA and ST data before intervention onset (baseline measurements). Data collection was performed from November 2013 until April 2019 with the exception of one year (October 2015 until December 2016) due to logistical reasons. The ActiGraph GT3X (Actigraph, Corp, USA) accelerometer was provided to 286 participants aged 4–18 years (89% of the total population) before the start of the lifestyle intervention. Children suffering from any musculoskeletal condition that would prevent the subject from performing PA or children that were wheelchair dependent did not receive an accelerometer. Fig. 1 provides an overview of the inclusion procedure of the study. Subsequently, both parents of all children gave written informed consent. Informed consent was also obtained from children aged ≥12 years. The study is registered at ClinicalTrial.gov (registration number: NCT02091544).

### Measurements

#### Accelerometry

The Actigraph GT3X is a triaxial accelerometer. Accelerometry currently represents the most accurate, inexpensive, and reliable method for objectively measuring both the amount and intensity of PA and ST, also in children and adolescents [19, 20]. The participants were asked to wear the accelerometer attached via a waistband on the right hip bone for seven consecutive days during waking hours, except during water activities (e.g. showering, swimming) and intensive contact sports (e.g. judo). For other sports (i.e. running, ball sports etc.) participants were instructed to wear the accelerometer. Accelerometry data were downloaded using 10s epochs using Actilife software (Actigraph, Corp, USA). Valid wear time was defined as a minimum of 4 days, consisting of at least 480 min per day of recording, including one weekend day. Derived data was expressed as mean counts per minute (cpm). To establish time spent in different intensity categories, the cut-off points developed by Evenson et al. (2008) were used; i.e. ST = 0–99 cpm, light intensity PA (LPA) = 100–2295 cpm, and MVPA ≥2296 cpm [21].

#### Anthropometrics

Anthropometric data were collected in the morning after an overnight fast, barefoot and wearing only underwear. Body mass was determined using digital scales (Seca, Chino, CA, USA) to the nearest 0.1 kg and height was measured to the nearest 0.1 cm using a digital stadiometer (De Grood Metaaltechniek, Nijmegen, The Netherlands). BMI (weight [kg] / height [m]^2^) was calculated and BMI z-scores were obtained using a growth analyzer (Growth Analyzer VE, Rotterdam, The Netherlands [22]), to adjust for age and sex. Children were categorized as overweight, obese or morbidly obese based on International Obesity Task Force (IOTF) criteria [23], corresponding to the 90th, 99th and 99.8th percentile respectively [24]. All anthropometric measurements were performed by trained health care personnel.

### Statistical analysis

Differences between groups (weight categories: overweight, obesity, morbid obesity; sex: boys, girls; age: < 12, ≥12 years) were assessed using ANOVA (with Fisher’s Least Significant Difference method for pair-wise comparisons if the overall test was significant) or independent-samples t-tests for numerical variables and chi-square tests for categorical variables. Correlations between PA and ST with BMI and age were determined using Pearson correlation coefficients (r).

Multivariable linear regression analyses were used to evaluate the associations between the dependent variable, i.e. one of the different PA intensities (total PA (cpm), light PA MVPA and ST) and the independent variables, i.e. weight categories (overweight, obese and morbid obese and therefore 2 dummy variables were created), sex (1 dummy variable), and age (numerical). As additional analyses, age was also dichotomized to distinguish primary school children (4–12 y) and secondary school children (≥12 y). Assumptions were checked using plots (scatterplots for linearity, P-P-plots and histograms for normality, residual plots for homoscedasticity), where Cook’s distance > 1 was used to define influential outliers. The sample size in the present study was large enough to perform linear regression analyses with the independent variables in the model, accounting for the rule of thumb that we need at least *n* = 10 per regression parameter.. As sensitivity analyses, the multivariable linear regression analyses were repeated for weekend- and weekdays separately. There was no missing data as the present study only involved baseline data and all measurements were performed on the same day, while an inclusion criterion was valid wear time, implying that PA and ST data are not missing. A *p*-value ≤ .05 was considered statistically significant. All analyses were performed using IBM SPSS Statistics for Windows version 25.0 (IBM Corp., Armonk, NY, USA).

## Results

A total of 202 children were eligible for this study, of which 29% presented with overweight, 46% with obesity and 25% with morbid obesity (Table 1). Supplementary file 1 shows the descriptives of the children who were included or excluded based on the wear time validation. Of the included children, boys and girls were equally distributed in all weight categories and in both age groups. The wear-time of the accelerometer was on average (± SD) 851 ± 132 min/day. There were no significant differences in wear-time between the different weight categories. Children spent on average 589 ± 142 min/day in ST which correspondents to 69% of the day (based on wear-time). In addition, children spent on average 221 ± 63 min/day of LPA per day and 41 ± 19 min/day of MVPA. Sixteen percent (*n* = 32) of the children reached the PA guideline of a minimum 60 min of MVPA per day. Due to the lack of specific recommendations on the amount of ST per day, the percentage of children who meet any ST guideline is not possible.

**Table 1:** Participant characteristics, PA levels and ST for the total sample as well as for the different weight categories, sex and age group

	Total ***N*** = 202	OV ***N*** = 58	OB ***N*** = 93	MO ***N*** = 51	P	B ***N*** = 90	G ***N*** = 112	P	Primary school age (<12y) ***N*** = 98	Secondary school age (≥12 y) ***N*** = 104	P
**Demographics**											
Age, years	12 ± 3	12 ± 3	12 ± 3	12 ± 4	.729	12 ± 3	12 ± 3	.222	9 ± 2	15 ± 2	**<.001**
Sex, M/F	45/55	50/50	43/57	41/59	.604				47/53	42/58	.530
**Anthropometry**											
BMI z-score	3.15 ± .73	2.39 ± .33 ^a, c^	3.16 ± .44^a,b^	4.00 .53^b, c^	**<.001**	3.3 ± .8	3.0 ± .7	.**010**	3.13 ± .73	3.17 ± .74	.701
**PA**											
Total PA, CPM	768 ± 298	780 ± 281	715 ± 263^b^	851 ± 357^b^	**.030**	861 ± 330	698 ± 253	**<.001**	910 ± 300	634 ± 227	**<.001**
ST, min/day	589 ± 142	601 ± 130^c^	606 ± 138^b^	547 ± 154^bc^	**.046**	572 ± 151	602 ± 134	.170	536 ± 133	640 ± 131	**<.001**
ST, %/day	69 ± 10	69 ± 9	70 ± 8^b^	66 ± 11^b^	**.033**	67 ± 11	70 ± 9	**.010**	65 ± 10	73 ± 8	**<.001**
LPA, min/day	221 ± 63	223 ± 63	214 ± 62	230 ± 65	.300	230 ± 68	213 ± 59	.073	248 ± 58	195 ± 57	**<.001**
LPA, %/day	26 ± 8	26 ± 7	25 ± 8^b^	29 ± 10 ^b^	**.048**	28 ± 9	36 ± 7	.056	30 ± 8	23 ± 6	**<.001**
MVPA, min/day	41 ± 19	44 ± 18	38 ± 18	43 ± 20	.106	47 ± 20	36 ± 16	**<.001**	44 ± 19	38 ± 18	**.025**
MVPA, %/day	5 ± 2	5 ± 2	4 ± 2	5 ± 3	.056	6 ± 3	4 ± 2	**<.001**	5 ± 3	4 ± 2	**.008**
Wear-time min/day	851 ± 132	868 ± 118	857 ± 137	820 ± 134	.139	851 ± 135	851 ± 130	.998	827 ± 126	873 ± 133	**.013**

Abbreviations: *OB* Obesity, *OV* Overweight, *MO* Morbid Obesity, *B* Boys, *G* Girls, *BMI* Body mass index, *PA* Physical activity, *CPM* Counts Per Minute, *ST* Sedentary time, *LPA* Light physical activity, *MVPA* Moderate-to-vigorous physical activity. Physical activity intensities are presented as mean minutes per day ± standard deviation or as percentage of wear time. ^a^ Statistical difference between overweight and obese children ^b^ Statistical difference between obese and morbidly obese children ^c^ Statistical difference between overweight and morbidly obese children

### Correlations between PA levels and ST with BMI z-score and age

Pearson correlations between PA and ST with BMI z-score and age are shown in Supplementary file 2. Higher total PA (cpm) was related to a lower BMI z-score (r = 0.158, *p* = 0.025) and less time spent in ST (min/day) was related to a lower BMI z-score (r = − 0.175, *p* = 0.013. In addition, total PA (cpm) was negatively correlated with increasing age (r = − 0.519, p = < 0.001) and ST (min/day) was positively correlated with increasing age (r = 0.411, p = < 0.001).

### Physical activity and sedentary time across weight categories, age and sex

Children with morbid obesity were significantly more physically active (851 ± 357 cpm vs 715 ± 263 cpm, *p* = .009) and less sedentary (547 ± 154 min/day vs 606 ± 138 min/day *p* = .018) compared to children with obesity (Table 1). Multiple linear regression shows that after correcting for age and sex, children with morbid obesity performed more total PA (cpm) (corrected difference (B) = 188, *p* = .006) and less ST (B = -51 *p* = .024) compared to children with obesity (Table 2). Boys were significantly more physically active (861 ± 330 cpm versus 698 ± 253 cpm, p = <.001) and performed more MVPA (47 ± 20 versus 36 ± 16 min/day, p = <.001) compared to girls. In addition, for each year increase of age, PA decreased on average with 46 cpm (p = <.001) and ST increased with 18 min/day (p = <.001). As additional analyses, primary school children (4–12 y) and secondary school children were distinguished (≥12 y). Primary school children were more physically active compared to secondary school children (910 ± 300 cpm versus 634 ± 227 cpm, *p* < .001).

**Table 2:** Results of the multiple linear regression analysis with PA levels and ST as the dependent variables and weight categories, age and sex as independent variables

	Overall ***p*** value between weight categories	OB vs OV B (95% CI) P	MO vs OV B (95% CI) P	MO vs OB B (95% CI) P	Sex (B vs G) B (95% CI) P	Age (per year) B (95% CI) P
Total PA, CPM	**.022**	−53 (− 134, 28) .195	65 (− 28, 158) .170	118 (34, 202) **.006**	132 (63,201) **< .001**	− 46 (− 57, − 35) **< .001**
ST, min/day	.057	2 (−40, 45) .917	−49 (− 98, 0) .050	−51 (− 95, − 7) .024	− 20 (− 56, 17) .287	18 (12, 23) **< .001**
ST % per day	**.029**	1 (− 1, 4) .385	−3 (− 6, 0) .095	−4 (− 7, − 1) **.008**	−3 (− 5, − 1) **.016**	2 (1, 2) **< .001**
LPA, min/day	.376	−9 (− 27, 10) .353	4 (− 17, 25) .702	13 (− 6, 32) .190	11 (− 5, 26) .164	−10 (− 12, − 7) **< .001**
LPA % per day	**.044**	−1 (− 3, 2) .604	2 (0, 5) .074	3 (1, 5) **.014**	2 (0, 3) .104	−1 (− 2, − 1) **< .001**
MVPA, min/day	.128	−5 (− 11, 1) .085	−1 (− 7, 7).980	5 (− 1, 11) .104	11 (6, 16) **< .001**	−1 (− 2, 0) **.041**
MVPA % per day	.062	−1 (− 1, 0) .125	0 (− 1, 1) .489	1 (0, 2) .026	1 (1, 2) **< .001**	0 (−0.2, − 0,04) **.008**
Wear-time min/day	.175	− 11 (− 54, 31) .599	−45 (− 94, 4) .073	−34 (− 78, 11) .140	2 (− 34, 39) .910	7 (1,13) **.014**

Abbreviations: *B* unstandardized regression coefficient (corrected effect), *OB* Obesity, *OV* Overweight, *MO* Morbid Obesity, *B* Boys, *G* Girls, *PA* Physical activity, *CPM* Counts Per Minute, *ST* Sedentary time, *LPA* Light physical activity, *MVPA* Moderate-to-vigorous physical activity. Physical activity intensities are presented as mean minutes per day ± standard deviation or as percentages

### Physical activity and sedentary time on week- and weekend day

After correcting for age and sex, children with morbid obesity perform more total PA (cpm) during weekdays (B = 122, *p* = .005) as well as during weekend days (B = 130, *p* = .030) compared to children with obesity (Table 3). In addition, for each year increase in age PA decreases both on weekdays (B = -45, p = <.001) and weekend days (B = -50, p = <.001). Furthermore, during weekdays children with morbid obesity spent less time sedentary (B- 72, *p* = 0.002) compared to children with obesity. During weekend days there was no significant difference between weight categories (Table 3).

**Table 3:** Results of the multiple linear regression analysis with PA levels and ST during week- and weekend days as dependent variables and weight categories, age and sex as independent variables

	Overall ***p*** value between weight categories	OB vs OV B (95% CI) P	MO vs OV B (95% CI) P	MO vs OB B (95% CI) P	Sex (B vs G) B (95% CI) P	Age (per year) B (95% CI) P
Total PA on weekday (CPM)	**.020**	−41 (− 123, 40) .319	81 (− 13, 175) .091	122 (37, 208) **.005**	150 (80,219) **< .001**	−45 (− 56, − 34) **< .001**
Total PA on weekend day (CPM)	.094	− 46 (− 159, 66) .419	84 (−46, 213) .203	130 (13, 247) .**030**	94 (−2, 190) .054	−50 (− 65, − 35) **< .001**
ST on weekday (min/day)	**.006**	10 (−34, 53) .663	−62 (− 112, − 13) .014	−72 (− 117, − 27) **.002**	−22 (− 58, 15) .246	19 (13, 24) **< .001**
ST on weekend day (min/day)	.393	19 (− 37, 75) .501	−21 (− 86, 44) .521	−40 (− 99, 18) .176	3 (− 45, 51) .895	17 (10, 25) **< .001**
LPA on weekday (min/day)	.154	−9 (− 28, 10) .375	11 (− 11, 33) .329	19 (−.378, 39) **.**054	13 (− 3, 30) .102	−10 (− 13, − 8) **< .001**
LPA on weekend day (min/day)	0.982	−1 (− 25, 23) .919	1 (− 26, 29).936	2 (− 23, 27) .853	8 (−13, 28) .456	−9 (− 12, − 6) **< .001**
MVPA on weekday (min/day)	.331	−4 (− 10, 2) .205	−1 (− 7, 7) .981	4 (−3, 10) .234	11 (6, 16) **< .001**	−1 (− 2, − 0,4) .063
MVPA on weekend day (min/day)	.136	−6 (− 14, 2) .117	1 (−8, 10) .837	−34 (− 78, 11) .140	9 (3, 16) **.006**	−1 (− 2, 0.07) .068

Abbreviations: *B* unstandardized regression coefficient (corrected effect), *OB* Obesity, *OV* Overweight, *MO* Morbid Obesity, *B* Boys, *G* Girls, *PA* Physical activity, *CPM* Counts Per Minute, *ST* Sedentary time, *LPA* Light physical activity, *MVPA* Moderate-to-vigorous physical activity. Physical activity intensities are presented as mean minutes per day ± standard deviation or as percentage

## Discussion

To our knowledge, this is the first study that evaluated objectively measured PA (total PA, light PA, MVPA) and ST using accelerometry in children across the different weight categories overweight, obesity and morbid obesity. The present study shows that children with morbid obesity performed in total more PA (cpm) than children with obesity. In addition, children with morbid obesity spent a lower percentage of time being sedentary and a higher percentage of time in LPA. The difference in total PA (cpm) between these two weight categories exists during both weekdays and weekend days. In addition, children with morbid obesity spent less ST during a week day compared to children with obesity.

Previous studies showed that children with obesity perform less PA and more ST compared to children with normal weight [15, 25]. Extending this finding to the subgroups of children with overweight, obesity and morbid obesity had led to our hypothesis that PA decreases and ST increases with increasing overweight severity in these subgroups. Although the correlation analyses of this study showed that higher PA and less ST were both related to a lower BMI z-score, more in depth analysis refuted the hypothesis. Multivariable linear regression analyses that evaluated the differences in PA and ST across the weight categories (i.e. overweight, obesity and morbid obesity) showed that children with morbid obesity perform more PA (cpm) and spent a lower percentage of time being sedentary compared to children with obesity Several explanations for this finding can be considered. First, children in the present study were excluded from the PA analysis if they did not reach the wear time criteria. The percentage of children excluded based on these criteria was higher in the obese and morbidly obese categories than in the overweight category. The results from the study of Schneller et al. (2017) showed that being less physically active and having a higher BMI percentile were indicators of a lower wear time [26]. Therefore, it is possible that a larger number of children with obesity and morbid obesity, who were less physically active, did not reach the wear time validation and hence influenced the results. However, given the similar dropout (31% versus 35% between the obese and morbidly obese group, it is unlikely that this would have affected the main conclusion. Another explanation for the differences between the weight categories could be that children with morbid obesity might be more aware of a healthy and active lifestyle than children with less severe obesity after being referred to the obesity center for treatment and might be more motivated to improve PA and reduce ST already before the start of the intervention. The results of the study from Taylor et al. (2014) showed that motivation (to change body weight and PA habits) was higher among parents from children with more severe overweight [27]. For the present study, it could be that the children with morbid obesity (and their parents) were more concerned about their health and/or experienced already mental or physical problems after being referred by the youth healthcare division or general practitioners. This could explain that these children already became more physically active before the start of the intervention as compared to children with overweight or obesity. In addition, the development of overweight or obesity is multifactorial and complex. Not only the amount of PA and ST, but also nutrition, metabolic, environmental, psychosocial, and cultural factors are considered to play a key role in obesity development and maintenance. For example, according to Nemet et al. (2010) food consumption increased after moderate intensity PA in children with overweight. However, food intake decreased after moderate intensity PA in children with normal weight [28]. Based on these findings, it could be suggested that even though children with morbid obesity were more physically active, they may compensate higher PA with a higher calorie intake. One could also question whether the degree of obesity may somehow affect the accuracy of the accelerometer to assess the amount of PA and ST. However, the Actigraph accelerometer, which was used in the present study, was shown before to measure activity counts equally accurate across different weight categories [29].

In agreement with previous studies that investigated PA levels and ST in the general population, the present study shows that boys are more physically active and spent less ST compared to girls and PA levels increase with age, up to an age of 10–11 years old, and then decrease at > 11 years when children head into puberty [30, 31]. Specifically, primary school-aged children (< 12 years) showed higher total PA and less ST compared to secondary school-aged children (≥12 years). The higher level of total PA in boys could be explained by a higher intrinsic motivation and experiencing more pleasure from exercise compared to girls [32]. Furthermore, previous studies found sex differences concerning different PA types [33, 34]. The results of the study of Reimers et al. (2018) showed that boys were more likely to engage in sports and active games, while girls prefer walking and/running or to play in a playground [34]. Therefore, it is recommended to provide tailored PA types in order to stimulate and improve PA and reduce ST. For example, a caregiver could inform and evaluate which specific PA types a child prefers and promote these activities in order to increase intrinsic motivation for PA [35]. Additionally, the negative association between PA and age and the positive association between ST and age highlights the importance of early PA promotion and reducing ST since the presence of comorbidities is already evident in primary school children with obesity [36]. A methodological strength of this study is the use of accelerometry to measure PA and ST in children across different weight categories (overweight, obesity and morbid obesity). Previous studies used self-reported PA or used objective methods but did not differentiate between weight categories. The present study also evaluated differences between PA levels and ST in boys and girls and in different age categories in children with overweight and (morbid) obesity. A limitation of the present study was that children were instructed to remove the accelerometer during water activities and some contact sports, which may have impacted the accelerometry data. This is common for accelerometer-derived data. The time spent on these activities is generally very small compared to the entire observation interval. Another limitation is that factors as food consumption and SES were not measured in the present study while these are established factors in the development of obesity. It is recommended to address these kind of factors in future research, especially in a larger study-group. Finally, there were differences between the weight categories regarding the number of children who were included and excluded in the PA analyses, based on the wear time validation. It is recommended to use skin-taped accelerometers to increase the compliance and adherence of the accelerometer. In this way, the potential risk of differences between groups will be decreased.

In the present paper cross sectional analysis are described before the start of a lifestyle intervention. Follow -up data of children participating in the COACH program are continuously being collected to determine the effect of the lifestyle intervention on the amount of PA and ST and changes therein across overweight categories including age and sex. The evaluation of PA and ST over time during this intervention will demonstrate whether PA and ST could be changed in the different subgroups.

## Conclusion

In conclusion, this cross-sectional study showed that children with obesity perform less PA and more ST (%/day) compared to children with morbid obesity. Secondly, PA decreased and ST increased with increasing age in all subcategories of overweight severity. Future research could explore the preferences and needs for PA guidance in children from different weight categories. This could consequently facilitate the development of effective interventions to motivate children to perform PA. In addition, longitudinal research with a representative sample is required to confirm the results of the present study.

## Supplementary Information

**Additional file 1.** Age and sex characteristics of children which are included or excluded for physical activity analyses based on wear time validation.

**Additional file 2.** Correlations between PA levels and ST with BMI and age.

## Data Availability

All data generated or analysed during this study are included in this published article.

## Abbreviations

COACH: Centre for Overweight Adolescent and Children’s Health Care
cpm: Counts Per Minute
FM: Fat mass
FFM: Fat free mass
LPA: Light physical activity
MVPA: Moderate to vigorous physical activity
PA: Physical activity
ST: Sedentary time
