# Supplementary material for: Objectively measured physical activity and sedentary time in children with overweight, obesity and morbid obesity: a cross-sectional analysis
Source: BMC Public Health. 2021 Aug 17;21:1558. doi: 10.1186/s12889-021-11555-5 (PMC8369633; doi:10.1186/s12889-021-11555-5)
Supplement: Supplementary file 1 — Additional file 1. Age and sex characteristics of children which are included or excluded for physical activity analyses based on wear time validation. [file 12889_2021_11555_MOESM1_ESM.docx]

**SUPPLEMENTARY FILE 1:**

**Age and sex characteristics of children which are included or excluded for physical activity analyses based on wear time validation**

|  | **Included in the analysis** | **Excluded from the analysis** | p-value |
| --- | --- | --- | --- |
| **Overweight** | **N = 58 (29%)** | **N=15 (18%)** |  |
| Age, years | 12 ± 3 | 12 ± 4 | 0.911 |
| Sex, M/F | 50/50 | 40/60 | 0.453 |
| **Obese** | **N=93 (46%)** | **N=42 (50%)** |  |
| Age, years | 12 ± 3 | 13 ± 3 | 0.151 |
| Sex, M/F | 43/57 | 60/40 | 0.079 |
| **Morbid obesity** | **N=51 (25%)** | **N = 27 (32%)** |  |
| Age, years | 12 ± 4 | 12 ± 4 | 0.873 |
| Sex, M/F | 41/59 | 63/37 | 0.110 |

Abbreviations: M Male, F Female.
