# Supplementary material for: Objectively measured physical activity and sedentary time in children with overweight, obesity and morbid obesity: a cross-sectional analysis
Source: BMC Public Health. 2021 Aug 17;21:1558. doi: 10.1186/s12889-021-11555-5 (PMC8369633; doi:10.1186/s12889-021-11555-5)
Supplement: Supplementary file 2 — Additional file 2. Correlations between PA levels and ST with BMI and age. [file 12889_2021_11555_MOESM2_ESM.docx]

**SUPPLEMENTARY FILE 2:**

**Correlations between PA levels and ST with BMI and age**

|  | **BMI z-score**  **r** | **Age**  **r** | |
| --- | --- | --- | --- |
| **Total PA, cpm** | 0.158* | -0.519* |  |
| **ST, min/day** | -0.175* | 0.411* |  |
| **ST % per day** | -0.159* | 0.533* |  |
| **LPA, min/day** | 0.066 | -0.494* |  |
| **LPA % per day** | 0.151* | -0.572* |  |
| **MVPA, min/day** | 0.081 | -0.175* |  |
| **MVPA % per day** | 0.130 | -0.215* |  |

Abbreviations: r Pearson correlation coefficient, PA Physical activity, CPM Counts Per Minute, ST Sedentary time, LPA Light physical activity, MVPA Moderate-to-vigorous physical activity. * p <0.05.
